# Synthetic metabolic engineering-a novel, simple technology for designing a chimeric metabolic pathway

**DOI:** 10.1186/1475-2859-11-120

**Published:** 2012-09-06

**Authors:** Xiaoting Ye, Kohsuke Honda, Takaaki Sakai, Kenji Okano, Takeshi Omasa, Ryuichi Hirota, Akio Kuroda, Hisao Ohtake

**Affiliations:** 1 Department of Biotechnology, Graduate School of Engineering, Osaka University, 2-1 Yamadaoka, Suita, Osaka, 565-0871, Japan; 2 PRESTO, Japan Science and Technology Agency (JST), 4-1-8 Honcho, Kawaguchi, Saitama, 332-0012, Japan; 3 Department of Life System, Institute of Technology and Science, The University of Tokushima, 2-1 Minamijosanjimacho, Tokushima, 770-8506, Japan; 4 Department of Molecular Biotechnology, Hiroshima University, 1-3-1 Kagamiyama, Higashi-Hiroshima, Hiroshima, 739-8530, Japan

## Abstract

**Background:**

The integration of biotechnology into chemical manufacturing has been recognized as a key technology to build a sustainable society. However, the practical applications of biocatalytic chemical conversions are often restricted due to their complexities involving the unpredictability of product yield and the troublesome controls in fermentation processes. One of the possible strategies to overcome these limitations is to eliminate the use of living microorganisms and to use only enzymes involved in the metabolic pathway. Use of recombinant mesophiles producing thermophilic enzymes at high temperature results in denaturation of indigenous proteins and elimination of undesired side reactions; consequently, highly selective and stable biocatalytic modules can be readily prepared. By rationally combining those modules together, artificial synthetic pathways specialized for chemical manufacturing could be designed and constructed.

**Results:**

A chimeric Embden-Meyerhof (EM) pathway with balanced consumption and regeneration of ATP and ADP was constructed by using nine recombinant *E. coli* strains overproducing either one of the seven glycolytic enzymes of *Thermus thermophilus*, the cofactor-independent phosphoglycerate mutase of *Pyrococcus horikoshii*, or the non-phosphorylating glyceraldehyde-3-phosphate dehydrogenase of *Thermococcus kodakarensis*. By coupling this pathway with the *Thermus* malate/lactate dehydrogenase, a stoichiometric amount of lactate was produced from glucose with an overall ATP turnover number of 31.

**Conclusions:**

In this study, a novel and simple technology for flexible design of a bespoke metabolic pathway was developed. The concept has been testified via a non-ATP-forming chimeric EM pathway. We designated this technology as “synthetic metabolic engineering”. Our technology is, in principle, applicable to all thermophilic enzymes as long as they can be functionally expressed in the host, and thus would be potentially applicable to the biocatalytic manufacture of any chemicals or materials on demand.

## Background

The use of renewable feedstocks as a starting material for the production of a wide range of value-added chemicals, the so-called “biorefinery”, has been one of the most outstanding issues in building of a sustainable society [1,2]. Considerable research effort has been exerted to improve the economy of current microbial-fermentation-based biorefinery processes. The optimization of the metabolic flux of microbial cells by enhancing the expression levels of desired genes and/or by depleting those of undesired ones has emerged as a powerful strategy to improve microbial cells, the concept so called “metabolic engineering” [3]. However, these attempts often suffer from flux imbalances as artificially engineered cells typically lack the regulatory mechanisms characteristic of natural metabolism [4]. One of the possible strategies to overcome this limitation is to avoid the use of living microorganisms and to use only enzymes involved in the metabolic pathway [5,6]. This *in vitro* production system would offer a number of potential advantages over the conventional fermentation-based production process, such as better process flexibility, elimination of tight transcriptional regulation, and easy optimization of production processes by altering enzyme levels. The absence of a culture medium can markedly simplify the isolation and purification of the product of interest. Moreover, the elimination of microbial growth and byproduct formation would allow us to obtain stoichiometrical conversions as well as an ability to perform thermodynamic predictions of production yield.

Welch and Scopes reconstructed a glycolytic pathway using individually purified yeast enzymes. The reconstructed pathway is capable of converting 1 M (18% [w/v]) glucose to ethanol within 8 h with nearly 100% molar yield [7]. In their work, they demonstrated that the imbalance of ATP, which impedes the complete conversion of glucose to ethanol, can be prevented by adding an excess amount of arsenate to the reaction mixture. Glyceraldehyde-3-phosphate (GAP) dehydrogenase (GAPDH) can accept arsenate instead of phosphate to form 1-arseno-3-phosphoglycerate, which is simultaneously broken down to arsenate and 3-phosphoglycerate (3-PG). A similar experiment using a cell-free extract of *Zymomonas mobilis* resulted in the conversion of 2 M glucose to 3.6 M ethanol [8]. The final ethanol concentration (nearly 20% [v/v]) was higher than any natural fermentation system can achieve. Nevertheless, little attention has been paid to the practical application of *in vitro* production systems mainly owing to the economical unprofitability of processes involving enzyme purification.

**Figure 1:**
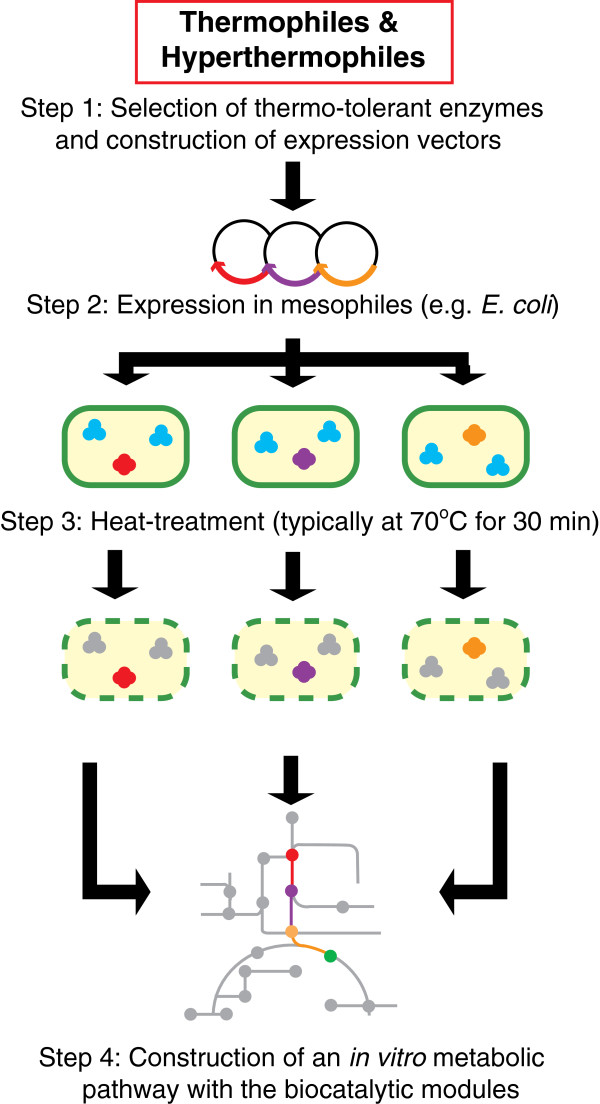
Schematic illustration of the basic procedure for synthetic metabolic engineering.

Thermophilic enzymes have recently been increasingly used in bioindustrial processes [9,10]. Recombinant DNA techniques allow the heterologous overproduction of thermophilic enzymes in mesophilic microorganisms (*e.g., Escherichia coli*). The use of recombinant mesophiles having thermophilic enzymes at high temperatures results in the denaturation of indigenous enzymes and the minimization of unwanted side reactions. Consequently, highly selective thermophilic biocatalytic modules comparative to the purified enzymes can be readily obtained without costly and cumbersome procedures for enzyme purification [11]. The rational combination of these biocatalytic modules makes it possible to construct *in vitro* synthetic metabolic pathways specialized for chemical manufacturing. More importantly, the excellent stability of thermophilic enzymes can mitigate the major disadvantage of *in vitro* enzymatic conversions, the inability of protein synthesis and renewal. We designated this novel, simple technology as “synthetic metabolic engineering”. To construct an artificial synthetic pathway by synthetic metabolic engineering, four key steps are included: 1) appropriate selection of thermostable enzymes; 2) expression in mesophilic hosts (*e.g.*, *E.coli*); 3) preheating of the cell suspension at high temperature (typically at 70°C for 30 min) to disrupt the cell membrane and to inactivate the indigenous host enzymes; and 4) rational combination of those catalytic modules at adequate ratio to achieve the stoichiometrical conversion (Figure 1).

As can be seen from Welch and Scope’s work, the prevention of cofactor depletion is a critical issue in constructing a synthetic metabolic pathway [7]. ATP and NAD(P)H are the most important biological phosphate and electron donor, respectively, as they are required for numerous enzymatic reactions in both anabolic and catabolic metabolisms. One of the possible strategies to prevent cofactor depletion is the integration of suitable cofactor-regeneration enzymes into a synthetic pathway. For instance, ATP regeneration systems using thermophilic polyphosphate kinase and polyphosphate have been developed and applied to the production of D-alanyl-D-alanine [12] and fructose 1,6-bisphosphate [13]. Thermophilic NAD(P)^+^-dependent 6-phosphogluconate dehydrogenase [14], glycerol dehydrogenase [15], and lactate dehydrogenase [16], are available for NAD(P)H regeneration. However, these cofactor-regeneration systems require an exogenous substrate serving as a phosphate and electron donor.

**Figure 2:**
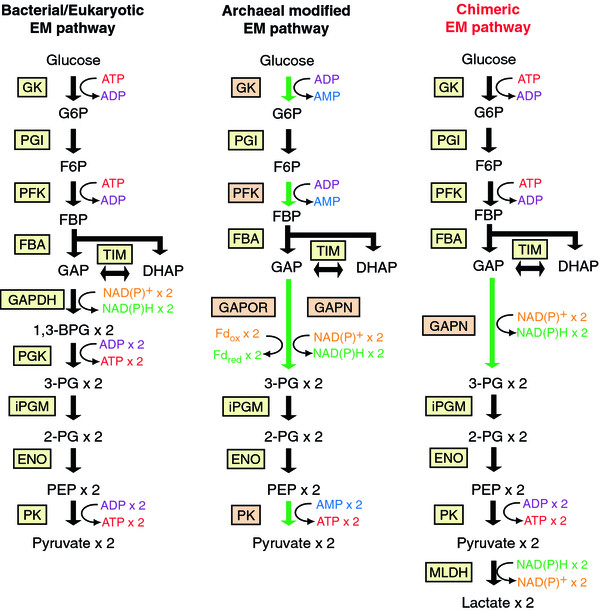
**Schematic illustration of design of chimeric Embden-Meyerhof (EM) pathway.** The bacterial/eukaryotic classic EM pathway (left), modified EM pathway of *Thermococcales* archaea (center), and chimeric EM pathway constructed in this study (right) are illustrated. Reactions catalyzed by enzymes uniquely involved in the archaeal modified EM pathway are indicated by green arrows. Abbreviations used: G6P, glucose-6-phosphate; F6P, fructose-6-phosphate; FBP, fructose-1,6-bisphosphate; GAP, glyceraldehyde-3-phosphate; DHAP, dihydroxyacetone phosphate; 1,3-BPG, 1,3-bisphosphoglycerate; 3-PG, 3-phosphoglycerate; 2-PG, 2-phosphoglycerate; PEP, phosphoenolpyruvate; GK, glucose kinase; PGI, glucose-6-phosphate isomerase; PFK, 6-phosphofructokinase; FBA, fructose-bisphosphate aldolase; TIM, triosephosphate isomerase; PGM, phosphoglycerate mutase; ENO, enolase; PK, pyruvate kinase; MLDH, malate/lactate dehydrogenase, GAPOR, glyceraldehyde-3-phosphate ferredoxin oxidoreductase; GAPN, non-phosphorylating glyceraldehyde-3-phosphate dehydrogenase; GAPDH, glyceraldehyde-3-phosphate dehydrogenase; PGK, phosphoglycerate kinase; Fd_ox_, oxidized ferredoxin; and Fd_red_, reduced ferredoxin.

Rapidly expanding genomics and metabolomics information has revealed a great diversity of microbial metabolisms. In particular, although the central metabolic routes of bacteria and eukaryotes are generally well-conserved, variant pathways consisting of several unique enzymes with unusual cofactor specificities have been developed in archaea [17]. For instance, the modified Embden-Meyerhof (EM) pathway of *Pyrococcus furiosus* has only four orthologues of the 10 glycolysis enzymes in bacteria and eukaryotes: triosephosphate isomerase (TIM), phosphoglycerate mutase (PGM), enolase (ENO), and pyruvate kinase (PK) [18]. Glucokinase (GK) and phosphofructokinase (PFK) of *P. furiosus* are ADP-dependent enzymes that are not related to the classical ATP-dependent kinases involved in the bacterial/eukaryotic EM pathway [19,20]. Although the conversion of GAP into 3-PG is catalyzed by the enzyme couple of the NAD^+^-dependent GAPDH and the ATP-generating phosphoglycerate kinase (PGK) in the classical EM pathway, the GAP ferredoxin oxidoreductase (GAPOR) is responsible for the single-step phosphate-independent conversion of GAP into 3-PG in *P. furiosus*[21]. A variant enzyme, non-phosphorylating GAPDH (GAPN), that utilizes NAD^+^ and/or NADP^+^ as the cofactor for the phosphate-independent oxidation of GAP has also been found and characterized in several archaeal strains involving *Thermoproteus tenax*[22], *Sulfolobus solfataricus*[23], and *Thermococcus kodakarensis*[24]. The substitution of GAPDH and PGK of the classical EM pathway with archaeal GAPN theoretically enables the construction of a chimeric EM pathway, in which the consumption and regeneration rates of ATP and ADP are balanced (Figure 2). Moreover, GAPN can bypass the production of the extremely thermolabile intermediate 1,3-bisphosphoglycerate [23]. Meanwhile, the glucose oxidation via the chimeric EM pathway yields the reducing equivalents in the form of NAD(P)H. To demonstrate the feasibility of the chimeric EM pathway, a NADH-dependent malate/lactate dehydrogenase was employed to achieve the redox balance of the cofactor and direct conversion from glucose to lactate.

Lactate has been attracting a great attention for its application in food, cosmetic, pharmaceutical and chemical applications [25]. However, in the conventional lactate fermentation process, fermentation broth contains impurities such as color, residual sugars, nutrients, and other organic acids, apart from cell mass [26]. Many studies concerning lactate separation using different techniques such as direct distillation, solvent extraction, adsorption and electrodialysis, have been conducted in order to reduce the operating cost [27]. Synthetic metabolic engineering without the use of culture medium would markedly simplify the isolation and purification of lactate.

In this work, the chimeric EM pathway was constructed by synthetic metabolic engineering using a mixture of nine different recombinant *E. coli* strains, each one of them overproducing one of seven glycolytic enzymes of *Thermus thermophilus*, the cofactor-independent PGM (iPGM) of *Pyrococcus horikoshii*, or the GAPN of *Thermococcus kodakarensis*. By coupling this chimeric EM pathway with the *T. thermophilus* malate/lactate dehydrogenase, a stoichiometric amount of lactate could be produced from glucose with an overall ATP turnover number of 31.

## Results

### Selection of enzymes for chimeric EM pathway

GAPN, a key enzyme for constructing a chimeric EM pathway, was derived from the hyperthermophilic archaeon *T. kodakarensis*. Although both NAD^+^ and NADP^+^ can serve as the electron acceptor, the *K*_m_ of *Thermococcus* GAPN for NADP^+^ is two orders of magnitude lower than that for NAD^+^[24]. However, the thermostability of NADP^+^ is considerably lower than that of NAD^+^, particularly under neutral and acidic conditions [28]. Owing to this fact, NAD^+^ was employed as the redox cofactor for the construction of a chimeric pathway. The enzyme can be strongly activated by the addition of glucose-1-phosphate (G1P) [24]. Under the assay conditions employed in this study, GAPN exhibited the highest specific activity in the presence of G1P at 100 μM or higher.

**Figure 3:**
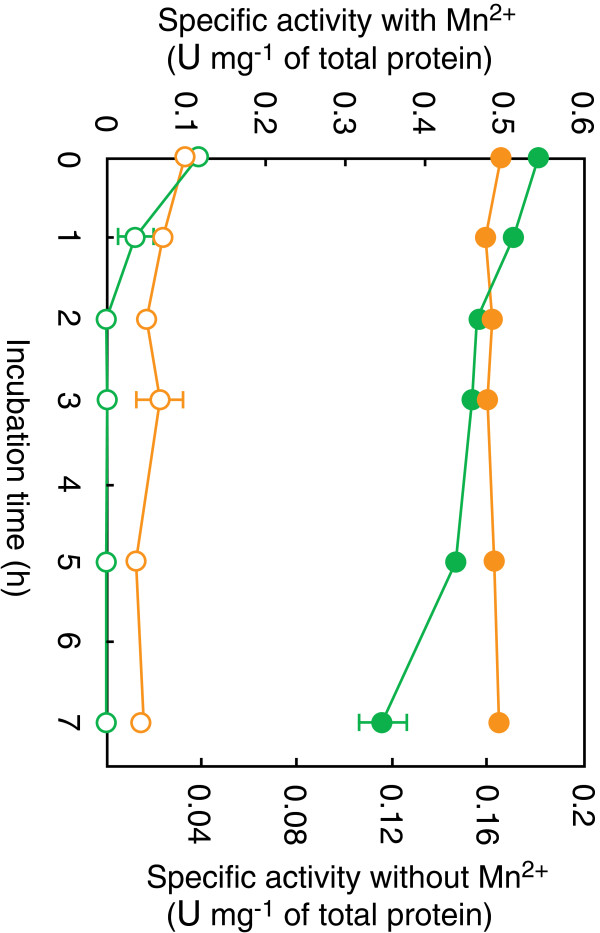
**Effects of Mn**^**2+**^**on the activity and thermal stability of the *Pyrococcus *iPGM and *Thermus *iPGM.** The crude extract of recombinant *E. coli* with *Pyrococcus* iPGM (orange circle) or *Thermus* iPGM (green circle) was incubated at 50°C in 50 mM HEPES-NaOH (pH 7.0) with (solid circle) or without (empty circle) 0.5 mM MnCl_2_. The residual iPGM activities were determined under standard assay conditions except that MnCl_2_ was absent in the assay mixture for the enzyme solutions incubated without MnCl_2_.

The thermophilic bacterium *T. thermophilus* HB8 was used as the source of other genes required for the construction of a chimeric pathway. Among them, the cofactor-independent phosphoglycerate mutase (iPGM) showed a relatively low thermal stability (Figure 3). Although the thermal inactivation of other enzymes was insignificant, the activity of the *Thermus* iPGM was almost completely lost after the incubation of the enzyme at 50°C for 2 h. We then isolated and heterologously expressed the gene encoding iPGM from a hyperthermophilic archaeon, *Pyrococcus horikoshii*, exhibiting a higher optimum growth temperature (98°C) [29] than that of *Thermus thermophilus* (70°C) [30]. Although the specific activity and thermal stability of the *Pyrococcus* iPGM were not markedly different from those of *Thermus* enzyme, they were considerably improved by the addition of Mn^2+^ (Figure 3). This finding was in good agreement with a previous report that the thermal stability of iPGM from the thermophilic archaeon *Archaeoglobus fulgidus*, which shares a 47.5% amino acid sequence identity with the *Pyrococcus* enzyme, can also be improved by an addition of Mn^2+^[31]. On the basis of these results, we employed *Pyrococcus* iPGM and used it in the presence of Mn^2+^.

**Figure 4:**
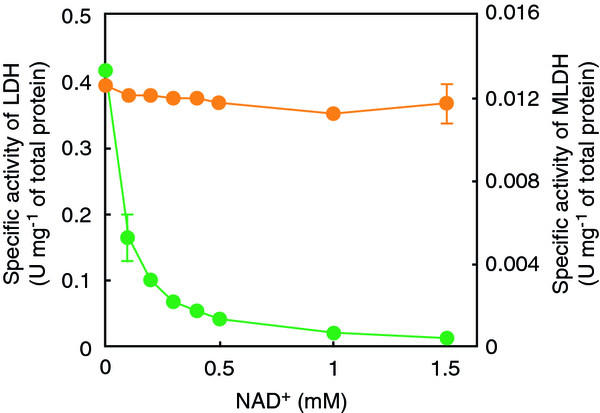
**Inhibitory effect of NAD**^**+**^**on activities of lactate dehydrogenase (green circle) and malate/lactate dehydrogenase (orange circle) of *T. thermophilus*.** The assay was performed at 50°C in the mixture composed of 50 mM HEPES-NaOH (pH 7.0), 0.2 mM pyruvate, 0.2 mM NADH, an appropriate amount of enzyme, and the indicated concentration of NAD^+^. After the preincubation for 2 min at 50°C, the reaction was initiated by pyruvate addition.

Some bacterial lactate dehydrogenases are inhibited by ATP and/or NAD^+^, and thus play an important role in the allosteric regulation of the flux of glycolysis [32]. Although the inhibitory effect of ATP (up to 1 mM) was almost negligible, the addition of 0.1, 0.2, and 1 mM NAD^+^ caused 60, 76, and 95% decreases in the activity of *Thermus* lactate dehydrogenase compared with that under the standard assay conditions, respectively (Figure 4). Owing to the low affinity of GAPN to NAD^+^, the chimeric pathway, therefore, required a certain amount of exogenous NAD^+^. This limitation led us to search for another enzyme that catalyzes the reduction of pyruvate to lactate. A putative NAD(P)H-dependent dehydrogenase, which was annotated as malate/lactate dehydrogenase (MLDH), was found in the gene-expression library of *T. thermophilus* HB8 [33]. The enzyme was confirmed to show pyruvate reducing activity with no inhibitory effect by NAD^+^ or other metabolic intermediates in this pathway (Figure 4). The natural substrate of this enzyme was unknown and it might more preferably accept a metabolite other than pyruvate in physiological conditions. However, the *in vitro* synthetic pathway involves only a limited number of necessary metabolites, and thus an enzyme with a broad substrate specificity is also available. More importantly, the allosteric regulation of the pathway flux can be eliminated by assembling enzymes derived from distinct organisms or metabolic pathways.

### Optimization of reaction conditions

**Figure 5:**
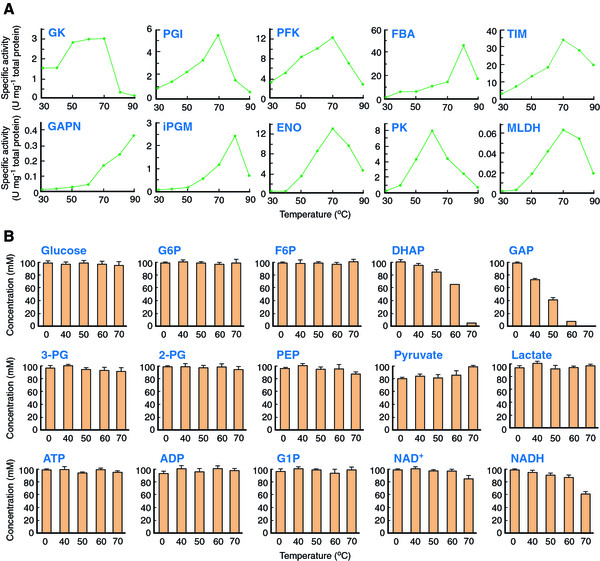
**Effects of temperature on enzyme activity and labilities of intermediates and cofactors involved in chimeric EM pathway.** (**A**) The enzyme activities were determined under the standard assay conditions (HEPES-NaOH, pH 7.0) at indicated temperatures. Specific activities were given as those in the crude extract of recombinant *E. coli* having each enzyme. One unit was defined as the amount of enzyme catalyzing the consumption of 1 μmol of the substrate per minute. (**B**) The intermediates and cofactors were dissolved in 50 mM HEPES-NaOH (pH 7.0) and incubated for 1 h at 0, 40, 50, 60, and 70°C. The residual concentration of intermediates was determined by CE-TOFMS [34]. ATP, ADP, NAD^+^, and NADH (0.1 mM) were determined as described in Materials and methods.

One of the major advantages of *in vitro* metabolic pathways is the flexibility of the reaction conditions. As long as the enzymes exhibit acceptable activities, reaction conditions can be freely adjusted and optimized. All the enzymes used for constructing the chimeric EM pathway exhibited their maximum activity at 60°C or higher (Figure 5A). However, the degradation of cofactors as well as the thermostabilities of some intermediates of EM pathway, particularly those of GAP, DHAP, and PEP, became significant at 60°C or higher (Figure 5B). Consequently, the reaction temperature of 50°C was chosen as a compromise between the thermostability of intermediates and enzyme activity. The specific activities of the crude extracts of *E. coli* recombinants harboring each enzyme were assessed at various pH (Table 1). On the basis of the pH profiles of each enzyme, the total protein concentrations of the enzyme mixture (containing 0.01 U each of GK, PGI, PFK, FBA, and TIM, along with 0.02 U each of GAPN, iPGM, ENO, PK, and MLDH) was found to be minimized at pH 7.0 (Table 1).

**Table 1:** Effects of pH on enzyme activity

**Buffer**	**pH**	**Specific activity (U mg**^**-1 **^**total protein)**^**a**^										**Total protein concentration (mg ml**^**-1**^**) **^**b**^
		**GK**	**PGI**	**PFK**	**FBA**	**TIM**	**GAPN**	**iPGM**	**ENO**	**PK**	**MLDH**	
MES	6	0.19	7.6	4.9	7.3	9.2	0.030	0.11	1.1	9.4	0.0026	8.6
MES	7	3.7	7.3	18	7.3	17	0.039	0.17	2.6	5.2	0.013	2.2
HEPES	7	2.9	2.3	8.8	6.9	13	0.036	0.20	3.1	4.5	0.019	1.7
HEPES	8	5.1	11	18	6.5	5.0	0.042	0.087	1.2	0.65	0.014	2.2
Bicine	8	4.5	16	15	4.0	2.1	0.035	0.078	1.3	1.0	0.011	2.7
Bicine	9	7.5	14	18	2.4	0.07	0.041	0.035	0.66	0.42	0.012	3.0
CHES	9	6.0	14	30	4.4	0.9	0.031	0.017	0.40	0.19	0.0095	4.1
CHES	10	2.6	6.1	10	4.6	2.3	0.0051	0.00075	0.10	0.047	0.0021	41

^a^ The specific enzyme activities in the crude extract of recombinant *E. coli* were determined under standard assay conditions (50°C) at indicated pHs.

^b^ The total protein concentration of the enzyme mixture (containing 0.01 U each of GK, PGI, PFK, FBA, and TIM, along with 0.02 U each of GAPN, iPGM, ENO, PK and MLDH) was estimated.

### Real-time estimation of production rate

For the real-time estimation of production rate, the whole pathway was divided in two parts; 1) the top part (glucose to 3-PG) and 2) the bottom part (3-PG to lactate). The former part, involving GK, PGI, PFK, FBA, TIM, and GAPN, catalyzed the conversion of glucose to 3-PG with concomitant NADH production, which could be spectrophotometrically monitored at 340 nm. On the basis of the enzyme activities, which were individually determined under standard assay conditions, essential units of enzymes (*i.e.,* 0.01 U each of GK, PGI, PFK, FBA, and TIM, and 0.02 U of GAPN) were incubated with 0.1 mM glucose at pH 7.0 and 50°C. The initial NADH production rate observed under these conditions, however, was considerably lower than the expected value of 0.02 μmol ml^-1^ min^-1^. The expected production rate could be achieved by increasing the units of PFK, FBA and GAPN to 0.2, 1 and 0.03 U, respectively. In the constructed pathway, the actual concentrations of the respective metabolites were kept at lower levels than those used in the standard assay conditions. Consequently, the reaction rates of each enzyme, especially those with relatively high *K*_m_ value for their substrates, were lower than those observed under standard assay conditions. Larger amounts of the enzymes were, therefore, required to achieve the expected production rate. Similarly, the NADH consumption rate of the bottom part (from 3-PG to lactate) was determined with an initial 3-PG concentration of 0.2 mM. The NADH could be consumed at a rate of 0.02 μmol ml^-1^ min^-1^ in the mixture containing 0.02 U each of iPGM, ENO, PK, and MLDH. By this way, the essential amounts of enzymes constituting the top and bottom parts of the synthetic pathway were experimentally determined.

### Lactate production by chimeric pathway

**Figure 6:**
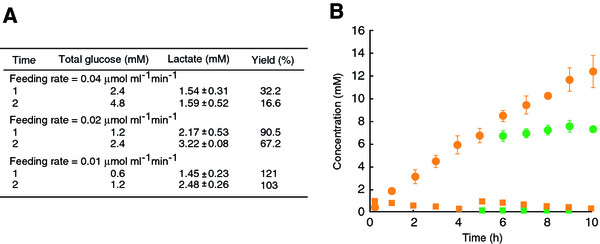
**Lactate production via chimeric EM pathway.** (**A**) The lactate production was performed as described in Materials and methods with glucose feeding at indicated rates. After the reaction for 1 and 2 h, lactate concentration was determined by HPLC, and yield on glucose (% mol/mol) was calculated. (**B**) Time course of lactate production at a glucose feeding rate of 0.01 μmol ml^-1^ min^-1^ (circle). The total concentration of NAD^+^ and NADH is indicated by squares. Lactate and NAD(H) concentrations with or without an additional NADH supplement (1 mM) after the initial 5-h reaction are shown by orange and green symbols, respectively.

All the enzymes employed in this study were heterologously produced in *E.coli* Rosetta2 (DE3) under control of the T7 promoter in the native form with no additional tag sequence. The recombinant cells were suspended in 50 mM HEPES-NaOH buffer (pH 7.0) and preheated at 70°C for 30 min to disrupt the cell membrane barrier and to inactivate *E. coli* enzymes. Lactate production using the chimeric pathway was performed directly using a mixture of heat-treated recombinant cells with the experimentally determined amounts of enzymes to achieve a production rate of 0.02 μmol ml^-1^ min^-1^. Addition of glucose to the reconstituted pathway at an inadequate dose was suspected to result in an uncontrolled glucose phosphorylation. As excess glucose feeding causes a rapid glucose phosphorylation by GK, insufficient ATP for the further phosphorylation of F6P catalyzed by PFK would be observed as a result. By contrast, lower feeding rate would result in a decrease in lactate production rate. Thus, unlike the use of living microbial cells, in which glucose uptake rate is regulated by specific glucose transporters [35,36], the optimization of substrate feeding rate is necessary for the operation of the reconstituted pathway. As we had expected, the molar yield of lactate production decreased when the feeding rate exceeded 0.01 μmol ml^-1^ min^-1^ (Figure 6A). The obtained yield which was slightly higher than theoretical yield was likely attributed to an addition of intermediates (0.1 mM glucose, 0.2 mM 3-PG and 0.2 mM pyruvate) into the reaction mixture, performed in order to achieve the constant production rate during the reaction. At a feeding rate of 0.01 μmol ml^-1^ min^-1^, production rate remained constant at its expected value (0.02 μmol ml^-1^ min^-1^) during the initial 5 h, and approximately 6 mM of lactate was produced from 3 mM of glucose (Figure 6B, orange circle). However, production rate started to decrease after 5 h, and the molar yield of lactate production rapidly dropped to 58% at 10 h (Figure 6B, green circle). Concurrently, pyruvate accumulation was detected. We observed that the thermal inactivation of all enzymes involved in the chimeric pathway was insignificant after the incubation at 50°C for 10 h. Meanwhile, the thermal decomposition of both NAD^+^ and NADH, particularly NADH, was not negligible (Figure 5B), and the depletion of the cofactor likely caused a decrease in the catalytic performance of MLDH. Moreover, the rate of the GAPN-mediated reaction was particularly sensitive to the depletion of the cofactor owing to its relatively high *K*_m_ for NAD^+^. A decrease in the rate of the GAPN-mediated reaction led to the accumulation and decomposition of the thermolabile intermediates GAP and DHAP, and to a decrease in the overall lactate production yield. Lactate production rate was recovered by addition of 1 mM NADH after the reaction for 5 h. The final lactate concentration reached 12 mM with an overall production yield of 100% at 10 h. From the actual ATP and ADP concentrations of the reaction mixture (0.4 mM), the ATP turnover number was calculated to be 31. The ATP turnover number was defined as moles of the product formed per mole of the cofactor added. The effect of cell-derived endogenous ATP and ADP (0.37 μM) was considered to be negligible.

## Discussion

Metabolic engineering has become a practical alternative to conventional chemical conversion particularly for biocommodity production processes; however, this approach is often hampered by as yet unidentified inherent mechanisms of natural metabolism. One of the current research directions in the field of metabolic engineering is to gain a deeper understanding of these underlying regulatory networks by exploiting the information obtained from a variety of “-omics” analyses [37]. By contrast, our approach, designated as “synthetic metabolic engineering”, provides a completely different means to overcome this limitation by reconstituting only the pathway of interest using thermo-tolerant biocatalytic modules. As well as being independent of transcriptional regulation, the assembly of enzymes derived from distinct organisms or metabolic pathways can eliminate the effect of allosteric regulation on the pathway flux.

It is vital to keep both energy and redox cofactors balanced to sustain overall reactions by synthetic metabolic systems because, unlike living biological systems, they are not equipped with complete enzyme apparatus to regenerate or resynthesize these cofactors. An injudicious “copy & paste” of natural pathways results in the depletion of specific cofactors since the physiological roles of metabolisms include the energy generation (catabolism) and energy-consuming synthesis of biomolecules (anabolism), in which the cofactors serve as a “currency” for transferring energy and redox power. In this work, we constructed a glycolytic pathway with no net ATP yield by chimerically integrating the archaeal GAPN to a classical EM pathway. The synthetic pathway produced a stoichiometric amount of lactate from glucose with an overall ATP turnover number of 31. Note that such a non-ATP-forming glycolysis pathway could no longer play a physiological role (*i.e.,* energy production particularly under anaerobic conditions) and thus would not be applicable for a fermentative purpose *in vivo*. In fact, although the GAPDH defect of *E. coli* can be complemented with the *Pisum sativum* GAPN under aerobic condition, this recombinant strain fails to grow anaerobically [38].

Although endogenous ATP regeneration was demonstrated in the present study, our results also indicated that the thermolability of NADH remained a major obstacle for the long-term operation of a synthetic metabolic system. A possible alternative to overcome this limitation is the replacement of NADH with more stable and low-cost artificial biomimetic NADH analogs [39]. Protein engineering approaches to improve the affinities of enzymes to these NADH biomimics may be also required [40]. Another important issue that should be resolved is the increase in production rate. Although it was predicted to increase proportionally along with a total enzyme concentration [41], the total amount of enzymes required to obtain a desired rate is often not practically achievable. On the other hand, naturally occurring cellular apparatus, in which a series of metabolic enzymes are packed in close proximity, can reduce the intermediate diffusion distance and therefore increase the overall reaction rate. The coexpression of thermophilic enzymes constituting a synthetic pathway in a single recombinant is an effective strategy to spatially organize the enzymes and achieve a high production rate [42].

## Conclusions

We proposed a novel and simple technology, designated as synthetic metabolic engineering and demonstrated its application to the construction of the non-ATP-forming chimeric EM pathway. The synthetic pathway produced a stoichiometric amount of lactate from glucose with an overall ATP turnover number of 31. The concept of *in vitro* synthetic-pathway biotransformation is not new but its feasibility in practical application has been largely restricted mainly owing to the prejudice that *in vitro* biotransformation is too costly for producing low-value biocommodities. However, the comparative cost analysis between *in vivo* and *in vitro* fermentation processes demonstrated that this interpretation is not necessarily true and that the development of stable standardized enzyme modules will provide economical advantages to the use of *in vitro* systems [5]. Synthetic metabolic engineering enables a one-step preparation of highly selective and stable biocatalytic modules via a simple heat-treatment of the recombinant mesophiles having thermophilic enzymes. Most importantly, it is, in principle, applicable to all thermophilic enzymes as long as they can be functionally expressed in the host, and thus would be potentially applicable to the biocatalytic manufacture of any chemicals or materials on demand.

## Materials and methods

### Bacterial strain and plasmid

The expression vectors for genes encoding GK, PGI, PFK, FBA, TIM, ENO, PK, and MLDH of *T. thermophilus* HB8 were obtained from the Riken *Thermus thermophilus* HB8 expression plasmid set [33]. The expression vector for GAPN [24] was a kind gift from Professor H. Atomi of Kyoto University. The gene encoding iPGM was amplified by PCR from the chromosomal DNA of *Pyrococcus horikoshii* OT3 (Takara Bio, Shiga, Japan) with the following primers: 5′-TTCATATGGTGCTAAAGAGGAAAGGC-3′ (the *Nde*I restriction site is underlined) and 5′-TTGAATTCTCAAGCTCCAAATTTTTCGCTCCT-3′ (the *EcoR*I restriction site is underlined). The amplified DNA was digested with *Nde*I and *EcoR*I and inserted into the corresponding restriction sites of pET21a (Novagen, Madison, WI, USA).

*E. coli* Rossetta2 (DE3) (Novagen) was used as a host strain for gene expression. The recombinants were aerobically cultivated in Luria-Bertani (LB) media containing 100 μg/ml ampicillin and 34 μg/ml chloramphenicol at 37°C. Gene expression was induced by an addition of 0.2 mM isopropylthiogalactoside (IPTG) to late-log culture for 4 h. Cells were harvested by centrifugation and resuspended in 50 mM HEPES-NaOH buffer (pH 7.0). The cell suspensions were heated at 70°C for 30 min before being used for lactate production.

### Enzyme assays

*E. coli* cell suspensions were disrupted with a UD-201 ultrasonicator (Kubota, Osaka, Japan), and the crude lysate was heated at 70°C for 30 min. Cell debris and denatured proteins were removed by centrifugation at 15,000 rpm and 4°C for 10 min. The supernatant was then used as an enzyme solution. Protein concentration was measured with the Bio-Rad assay system (Bio-Rad, Hercules, CA, USA) using bovine serum albumin as the standard.

One unit of an enzyme was defined as the amount consuming 1 μmol of the substrate per min under the assay conditions. The standard assay mixture for GK was composed of 50 mM HEPES-NaOH (pH 7.0), 0.1 mM glucose, 0.2 mM ATP, 5 mM MgCl_2_, 0.5 mM MnCl_2_, 1 mM NAD^+^, 1 mM G1P, 0.08 U of PGI, 0.2 U of PFK, 1 U of FBA, 0.1 U of TIM, 0.02 U of GAPN, and an appropriate amount of GK. The mixture without glucose was preincubated at 50°C for 2 min, and then the reaction was initiated by an addition of 0.1 mM glucose. The reduction of NAD^+^ was monitored at 340 nm using a UV-VIS spectrophotometer (Model UV-2450, Shimadzu, Kyoto, Japan). Similarly, the activities of PGI, PFK, FBA, TIM, and GAPN were spectrophotometrically assessed in the mixture containing the substrate for each enzyme (0.1 mM of glucose-6-phosphate, fructose-6-phosphate, fructose-1,6-bisphosphate, dihydroxyacetone phosphate, or 0.2 mM GAP, respectively) instead of glucose.

iPGM activity was assayed at 50°C in a mixture consisting of 50 mM HEPES-NaOH (pH 7.0), 0.2 mM 3-phosphoglycerate, 5 mM MgCl_2_, 0.5 mM MnCl_2_, 0.2 mM ADP, 0.2 mM NADH, 0.5 U of ENO, 0.5 U of PK, 1.2 U of LDH, and an appropriate amount of enzyme. ENO and PK were assayed in the same manner using 0.2 mM of 2-phosphoglycerate and phosphoenolpyruvate as the substrate, respectively.

The activities of LDH and MLDH were assessed at 50°C by mixing the enzymes with 50 mM HEPES-NaOH (pH 7.0), 5 mM MgCl_2_, 0.5 mM MnCl_2_, 0.2 mM NADH, and 0.2 mM pyruvate. NADH oxidation was monitored at 340 nm.

The heat-treated cell lysate of the recombinant *E. coli* harboring an empty vector showed no detectable level of enzyme activity under the assay conditions.

### Lactate production

The reaction mixture (4 ml) was composed of 0.1 mM glucose, 0.2 mM 3-PG, 0.2 mM pyruvate, 5 mM MgCl_2_, 0.5 mM MnCl_2_, 0.2 mM ATP, 0.2 mM ADP, 1 mM NAD^+^, 0.2 mM NADH, 1 mM G1P, and 50 mM HEPES-NaOH buffer (pH 7.0). The cell suspensions of *E. coli* producing GK, PGI, PFK, FBA, TIM, GAPN, iPGM, ENO, PK, and MLDH were preheated at 70°C for 30 min and then added into the reaction mixture at final concentrations of 2, 1, 1, 1, 1, 26, 3, 3, 3, and 100 mg (wet weight cells)/ml, respectively. The reaction mixture was stirred in a container kept at 50°C, and glucose solution (40 mM) was added to the mixture at a flow rate of 1 μl min^-1^ (= 0.01 μmol ml^-1^ min^-1^) using a Shimadzu LC-20 AD solvent delivery unit. Aliquots (50 μl) of the reaction mixture were withdrawn at 1 h intervals, diluted fourfold with distilled water, and centrifuged to remove the cell debris (15,000 rpm, 10 min). The supernatant was then ultrafiltered using Amicon 3 K (Millipore) and analyzed by a high-performance liquid chromatography (HPLC).

### Analytic methods

Lactate and pyruvate were quantified by HPLC on two tandemly connected ion exclusion columns (Shim-pack SPR-H, 250 mm × 7.8 mm, Shimadzu). The columns were eluted at 50°C using 4 mM *p*-toluenesulfonic acid as a mobile phase at a flow rate of 0.3 ml min^-1^. The eluent was mixed with a pH-buffered solution (16 mM Bis-Tris, 4 mM *p*-toluenesulfonic acid, and 0.1 mM EDTA) supplied at a flow rate of 0.3 ml min^-1^, and then analyzed for lactate using a conductivity detector (CDD-20A, Shimadzu). NAD^+^ and NADH concentrations were analyzed colorimetrically using a NAD/NADH quantification kit (Biovision, Mountain View, CA, USA) in accordance with the procedure provided in the kit. ATP and ADP concentrations were assessed quantitatively using the EnzyLight ADP/ATP ratio assay kit (BioAssay Systems, Hayward, CA, USA) according to the manufacturer’s instructions.

## Competing interests

The authors declare that they have no competing interests.

## Authors’ contributions

XY performed the experiments and wrote the manuscript. KH designed all the experiments and wrote the manuscript. TS, KO and RH co-performed the experiments on the enzyme characterization. TO and AK contributed general advice, particularly on the thermophilic microorganisms, and resource support, as well as edited the manuscript. HO conceived the project and wrote the manuscript. All authors read and approved the final manuscript.
